# Health care cost-containment measures in the context of the economic crisis: impact analysis

**DOI:** 10.1186/1750-1172-9-S1-O21

**Published:** 2014-11-11

**Authors:** François Houÿez, Ludovic Tessier, Dimitrios Synodinos

**Affiliations:** 1 European Organisation for Rare Diseases, Paris, France; 2 University of Southern Brittany, Lorient, France; 3 European Organisation for Rare Diseases Board of Directors and Greek Alliance for Rare Diseases (PESPA), Athens, Greece

## Aims

As a response to the economic and financial crisis that hit Europe and the rest of the world in 2008, most health authorities adopted series of measures to contain or to reduce the healthcare expenditure. In 2010-2011, some 116 health reforms were adopted or planned in the European Union (EU)/European Economic Area (EEA). Some measures can potentially severely impact public health. This analysis explores the impact of such measures for patients with rare diseases (RD).

## Methods

A literature search was done, in addition to the collection of information from RD patients’ organisations. Press articles as well as report from international organisations (OECD, EC, WHO) were analysed. Proceedings from conferences on the impact of the economic crisis on the health of citizens were used. There are important limits when analysing measures taken and the impact we observe: no causal relation can be made.

## Results

In many countries, the most frequent measures adopted to reduce the health consisted in price reduction of pharmaceuticals (15 price reductions in 11 countries), change in co-payment (13 measures in 9), reimbursement (8 countries), reference price system (10 countries) and INN prescribing made mandatory. Other health budgets were also affected (public health, care…).

Information on the consequences for citizens or patients is limited: increased incidence of suicides in the UK, of HIV infection cases in I.V drug users in Greece, with also an increase of stillbirths. For RD, according to one survey among 403 Greek patients, those with a RD were more likely to report a medicine shortage than patients with a chronic disease (37/96 versus 23/207). In France, reimbursement of transport to centres of expertise can be refused. In Romania, a list of 101 medicines for which a reimbursement decision has been postponed for 5 years includes 21 medicines for RD. In Spain, a list of 43 medicines that can be obtained at the hospital but now with co-payment includes 13 products for RD. In the UK, the fund for new treatments for RD was suppressed. In Germany, the price negotiation for most orphan medicinal products (OMP) with G-BA lasts for more than 15 months. A systematic review of articles on payer assessment for OMP showed that in average 1.4 articles were published by year between 2000 and 2008 and in average 8.8 between 2008 and 2013, indicating more acute difficulties with OMPs since the onset of the economic crisis.

## Conclusions

Our analysis certainly reveals increased access to care difficulties, with some indications of moderately severe issues but no global “catastrophe”. Facts and testimonies from literature search and patients are few, and the health impact of the measures can hardly be estimated. Our next action will consist of a questionnaire sent directly to patients with RD in Europe to document on the difficulties they are facing.

